# Deubiquitinating Enzymes Related to Autophagy: New Therapeutic Opportunities?

**DOI:** 10.3390/cells7080112

**Published:** 2018-08-19

**Authors:** Anne-Claire Jacomin, Emmanuel Taillebourg, Marie-Odile Fauvarque

**Affiliations:** 1 School of Life Sciences, University of Warwick, Coventry CV4 7AL, UK; 2 Biosciences and Biotechnology Institute of Grenoble, (CEA-DRF-BIG-BGE), Univ. Grenoble Alpes, INSERM U1038, CEA, F-38000 Grenoble, France; emmanuel.taillebourg@cea.fr

**Keywords:** autophagy, DUB, deubiquitinating enzymes, inhibitor, aggrephagy, mitophagy, chaperone-mediated autophagy, small-molecule, ubiquitin, ubiquitin proteases

## Abstract

Autophagy is an evolutionary conserved catabolic process that allows for the degradation of intracellular components by lysosomes. This process can be triggered by nutrient deprivation, microbial infections or other challenges to promote cell survival under these stressed conditions. However, basal levels of autophagy are also crucial for the maintenance of proper cellular homeostasis by ensuring the selective removal of protein aggregates and dysfunctional organelles. A tight regulation of this process is essential for cellular survival and organismal health. Indeed, deregulation of autophagy is associated with a broad range of pathologies such as neuronal degeneration, inflammatory diseases, and cancer progression. Ubiquitination and deubiquitination of autophagy substrates, as well as components of the autophagic machinery, are critical regulatory mechanisms of autophagy. Here, we review the main evidence implicating deubiquitinating enzymes (DUBs) in the regulation of autophagy. We also discuss how they may constitute new therapeutic opportunities in the treatment of pathologies such as cancers, neurodegenerative diseases or infections.

## 1. Introduction

Autophagy is a lysosomal catabolic process that ensures the degradation and recycling of intra-cytoplasmic components and, therefore, highly contributes to the maintenance of cellular homeostasis. In order to adapt to various stresses and promote its survival under challenging conditions, the cell also uses autophagy to degrade a broad range of endogenous or exogenous substrates [1]. The best-studied endogenous substrates of autophagy are mitochondria and protein aggregates [2,3,4]. Defects in the elimination of such substrates are often associated with human pathologies, including neurodegenerative diseases or cancers [5,6,7].

Autophagy is a very dynamic process and has to be tightly regulated to provide a timed and efficient response to a multitude of signals. Posttranslational modifications play a significant role in the induction and regulation of autophagy [8,9]. Ubiquitination of substrates and protein of the autophagy machinery has notably emerged as a central regulatory mechanism of autophagy acting at various levels to promote autophagy induction or shutdown [10,11].

Ubiquitination is a reversible protein modification that consists of the covalent attachment of one or several ubiquitin moieties to protein substrates [12,13,14]. While ubiquitination is achieved by the sequential action of E1 ubiquitin-activating enzyme, E2 ubiquitin-conjugating enzyme, and an E3 ubiquitin ligase, the removal of ubiquitin from a protein is catalyzed by deubiquitinating enzymes (DUBs) [15]. Ubiquitination can promote or interfere with protein-protein interactions, modifying the conformation or the activity of the targeted protein or direct proteins for degradation by either the proteasome or the autophagy process [16,17]. Ubiquitin linkage is involved in most, if not all, cellular processes and signaling pathways. As for autophagy, defects in ubiquitination and deregulation of DUBs are associated with human disorders [18,19,20].

Here, we chose to focus on the role of DUBs related to the regulation of autophagy and cargo lysosomal degradation. We extended our review to the recent development of small-molecule inhibitors modulating DUBs’ activity on autophagy, thus highlighting their potential as therapeutic targets.

## 2. Autophagy and Ubiquitin System: A Brief Overview

### 2.1. Autophagy

Autophagy was first described in 1966 by C. de Duve and R. Wattiaux based on observations from mammalian cells deprived of serum and amino acids [21]. The name autophagy was coined later, after electron microscopy observations that revealed the presence of double-membrane vesicles containing part of the cytoplasm. Autophagy can be induced in response to various stressors such as lack of nutrients or growth factors, hypoxia, or infection [22]. As such, autophagy was initially described as a survival mechanism in response to various stresses. However, it is now widely recognized that basal levels of autophagy also operate independently of any stress, contributing to the maintenance of cellular homeostasis by the removal of dysfunctional or unnecessary proteins and organelles (for instance, mitochondria) and ribosomes [2].

**Figure 1:**
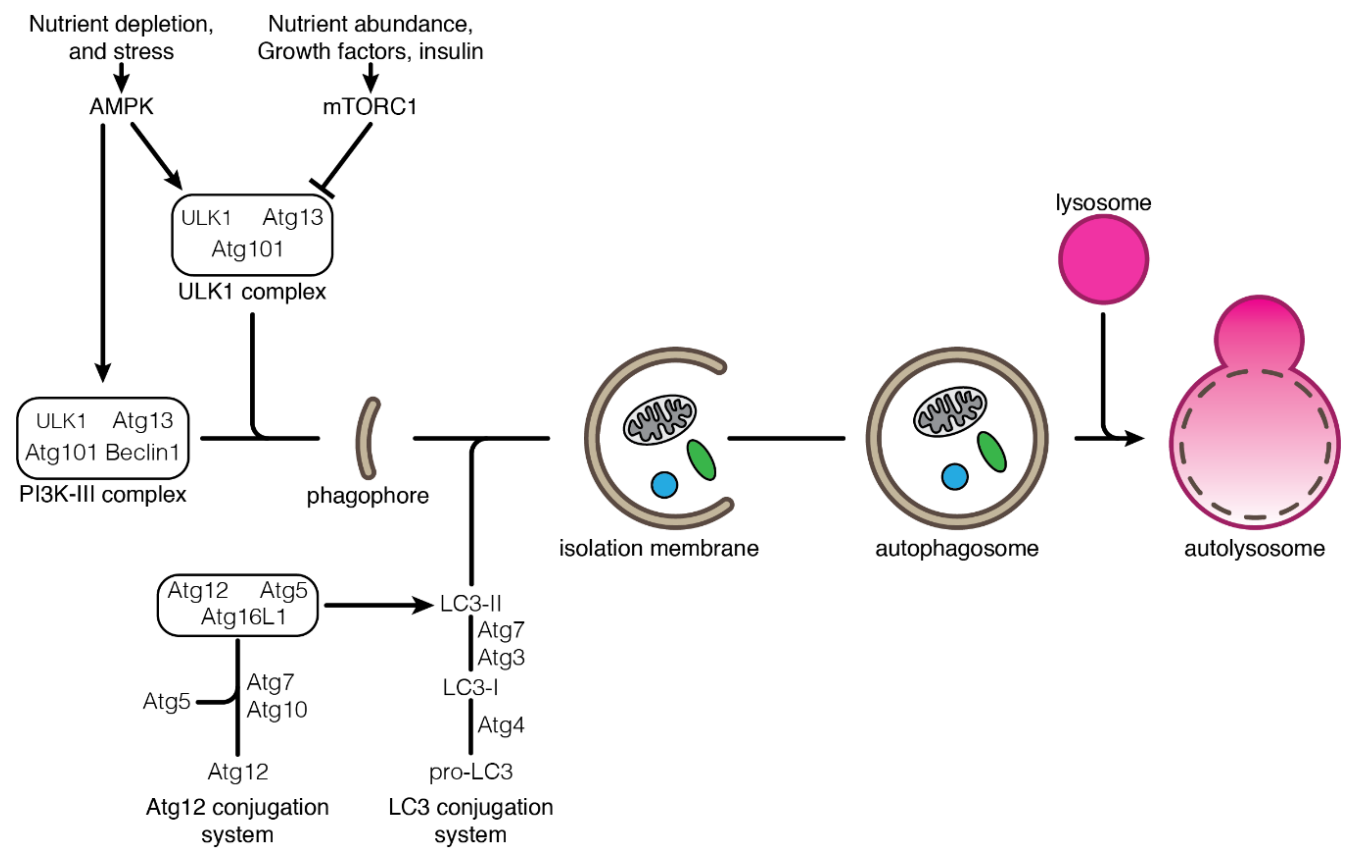
An overview of the macroautophagy process. Initiation of autophagy is under the control of sensor pathways that can sense the availability of nutrients, growth factors, insulin or other stresses. The main regulators of autophagy are AMPK (AMP-activated kinase) and mTORC1 (mTOR complex (1) which act as autophagy activator and inhibitor, respectively. AMPK and mTORC1 regulate the activation of the ULK1 complex which, together with the PI3K-Ш complex, initiates the autophagosome formation. The formation of an autophagosome starts with the generation of a phagophore which elongates into an isolation membrane where the cargos to be degraded are gathered. The enclosure of the isolation membrane forms a double-membraned autophagosome that matures and eventually fuses with the lysosome, forming an autolysosome where lysosomal hydrolases degrade its content. The Atg12 and LC3 conjugation systems are essential for the autophagy process and formation of the autophagosome. The Atg12 conjugation system consists in the formation of the Atg12-Atg5-Atg16L1 complex that then promotes the conjugation of LC3; pro-LC3 is cleaved by Atg4 (LC3-I) which is then conjugated with phosphatidylethanolamine (PE) (LC3-II) before being anchored to the nascent autophagosomal membrane.

One can distinguish three types of autophagy: macroautophagy (mostly referred to as autophagy), chaperone-mediated autophagy and microautophagy. These autophagy processes differ by the way substrates are being delivered to the lysosome for degradation. In macroautophagy, autophagy substrates are isolated from the cytoplasm through their engulfment in double-membrane vesicles called autophagosomes [23]. Autophagosomes are formed by the elongation of an isolation membrane derived from the endoplasmic reticulum [24]. The autophagosomes eventually mature and fuse with the lysosomes where hydrolases degrade the substrates (Figure 1). In chaperone-mediated autophagy, soluble cytosolic proteins are recruited by the chaperone HSC70/HSP8A and directly translocated into the lysosome through pores made of LAMP2A multimers. Substrate translocation into the lysosome requires a second HSC70 chaperone resident in the lysosomal lumen [25,26,27]. In microautophagy, cytosolic substrates are transferred to the lysosome through direct invagination of the lysosomal membrane (in yeast) or from the late endosomes (in mammals and *Drosophila*, referred to as endosomal-microautophagy) that eventually fuse with the lysosomes [28,29,30]. As for chaperone-mediated autophagy, endosomal-microautophagy requires the chaperone protein HSC70 for the selection of the substrates [28,30,31].

The major components of the autophagy machinery are the ATG (autophagy-related gene) proteins, which are required for the formation and maturation of the autophagosome (Figure 1). ATG proteins form specific macromolecular complexes acting at several steps of autophagosome formation and regulating different stages of the autophagy process [23]. Autophagy induction by starvation is controlled by the TOR (target of rapamycin) complex 1, which is an essential signaling pathway for the sensing of nutrient availability [32]. Lack of nutrients leads to the down-regulation of TOR activity, resulting in the activation of the ULK1/ATG1 complex. The activation of the ULK1 complex then initiates autophagy by recruiting other ATG proteins to the nascent autophagosome. Downstream ULK1 complex, the class III PtdIns3K (PI3K-III) complex, is responsible for the production of phosphatidylinositol-3-phosphate (PtdIns3P), which triggers the proper localization and membrane association of other ATG proteins [33]. Beclin1, a multivalent adaptor, is one major component of the PI3K-III complex. The expansion of the autophagosomal membrane requires two ubiquitin-like conjugation cascades: the Atg12-conjugation system and the Atg8/LC3-conjugation system. The modification of the Atg8 protein by lipidation and its anchorage in the autophagosome membrane is a hallmark of autophagy induction [34,35,36]. After completion and closure, the autophagosome is targeted to and fuses with the lysosome, leading to the degradation of its content. Posttranslational modifications on autophagy regulators contribute to the rapid and efficient response to various stresses. If attention is focused first on phosphorylation, a growing body of evidence shows that ubiquitination also plays a crucial role in the dynamic regulation of autophagy notably by targeting substrates to autophagosome, and modifying the activity of ULK1 and PI3K-III complexes [11,37,38].

### 2.2. Ubiquitin System

Ubiquitin is a small globular protein with a β-grasp superfold conformation [39,40]. Ubiquitin is covalently conjugated by its terminal glycine (G76) onto a lysine residue of a substrate protein. Protein ubiquitination is a complex process requiring the successive activity of three types of enzymes: an E1 ubiquitin-activating enzyme, an E2 ubiquitin-conjugating enzyme, and an E3 ubiquitin-ligase enzyme. The ubiquitination process can be broken down in two main steps: (1) The ATP-dependent activation of a ubiquitin molecule by conformational modification of its C-terminus extremity by an E1 enzyme followed by its transfer onto an E2 enzyme, and (2) the conjugation of the activated ubiquitin onto a substrate protein, mediated by an E3 enzyme that bridges the E2 to a specific substrate allowing for the subsequent transfer of ubiquitin from the E2 enzyme to the substrate through the formation of an isopeptide bond [41,42]. Alternatively, E3 ligases of the HECT family possess E2 and E3 activities [43,44]. There is also evidence for the addition of ubiquitin moieties onto non-lysine residues. The ubiquitination of cysteine or serine and threonine residues requires the formation of thiol- or oxy-ester bonds respectively [45].

Ubiquitination was discovered in 1980 and first described for its essential role in targeting proteins to the proteasome for degradation [46]. It is now widely recognized that ubiquitination also acts in other cellular processes. Indeed, a broad range of types of ubiquitination have been reported. Substrates can be modified by the attachment of a single ubiquitin molecule (mono-ubiquitination) or several single ubiquitin molecules on different lysine residues of the substrate (multi-mono-ubiquitination). Mono-ubiquitination is primarily described for its function in endocytosis of plasma membrane receptors [47]. Alternatively, several ubiquitin molecules can be ligated to one another using ubiquitin internal lysine residues, forming chains of ubiquitin moieties that can elongate on the substrate or directly be attached to a target protein (poly-ubiquitination). Because ubiquitin has seven lysine residues, there can be at least as many types of ubiquitin chains that can be generated (K6, K11, K29, K48, K63-linked ubiquitin chains) [48,49]. Poly-ubiquitin chains can also be assembled through N- to C-terminal interaction to form linear chains (M1) [50,51]. The tridimensional structures of ubiquitin chains vary depending on the lysine in the ubiquitin used to generate the chain and affect the function or stability of the substrate. For instance, K48-linked ubiquitin chains have a compact conformation and target substrates for proteasomal degradation, while K63-linked ubiquitin chains display an open conformation and are mostly described to promote signal transduction through the assembly of large protein complexes [52,53].

**Figure 2:**
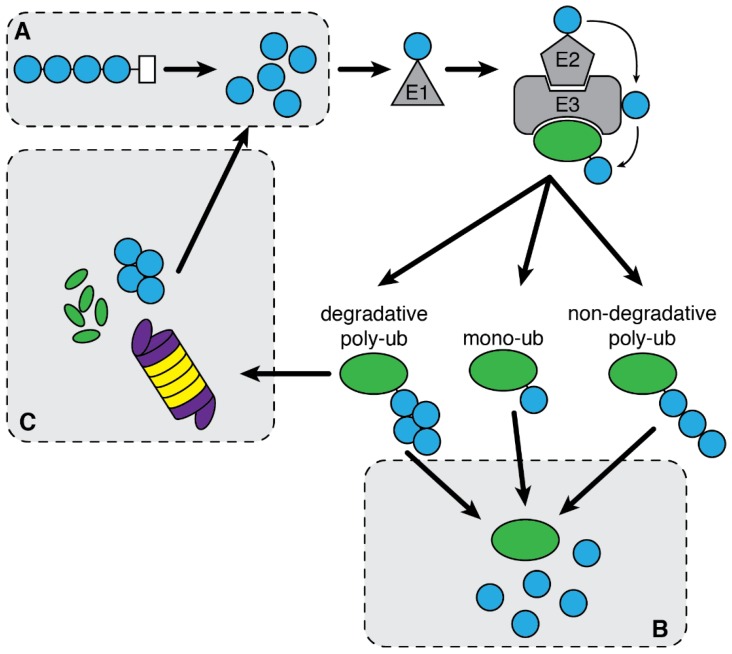
Localization of the action of DUBs in the ubiquitination process. Ubiquitination is catalyzed by three types of enzymes: E1, E2, E3. It is a reversible reaction. DUBs process ubiquitin chains at three level: (**A**) For the generation of ubiquitin monomers from a ubiquitin precursor; (**B**) for the selective removal of ubiquitin moieties on ubiquitinated proteins; and (**C**) for the recycling of ubiquitin from protein degraded by the proteasome.

Protein ubiquitination is a reversible posttranslational modification. The hydrolysis of ubiquitin linkages is conducted by a specific family of proteases: the DUBs. These enzymes can act at different stages of the protein ubiquitination process: (1) At the “initial” stage, by cleaving the ubiquitin precursors to supply ubiquitin monomers to the ubiquitination enzymes; (2) at the “intermediate” stage, by the regulated removal of ubiquitin moieties from proteins to alter their fate (stabilization, conformational change); and (3) at the “final” stage by the removal of ubiquitin chains from substrates addressed to the proteasome to facilitate their degradation and processing into ubiquitin monomers, free to enter a new ubiquitination cycle (Figure 2) [54,55,56]. The hydrolysis of K48-linked ubiquitin chains, most well-known to induce the proteasomal degradation, can affect the fate of the protein they are added to either by protecting substrates from degradation or by supporting proteasomal degradation, as the removal of K48-linked ubiquitin chains, mostly by proteasomal DUBs, is also required for protein entry into the proteasome [57,58].

There are approximately 100 DUBs encoded by the human genome [15,59], which are divided into two main families: the cysteine proteases and the metalloproteases. There are 12 DUBs from the metalloprotease family characterized by a JAMM (JAB1/PAB1/MPN domain-containing metalloenzymes) domain that catalyzes the hydrolysis of isopeptide bonds in the presence of Zn^2+^. Cysteine proteases are divided into five sub-families according to the sequence and structure of their catalytic domain: ubiquitin-specific proteases (USPs), ubiquitin C-hydrolases (UCHs), otubain proteases (OTUs), Machado Joseph disease proteases (MJDs), and the most recently identified sub-family MIU-containing novel DUB family (MINDYs). The most abundant sub-family of DUBs is the USPs with over 50 members, come after the OTUs (18 members), UCH, MJDs and MINDYs (each with four members) [15].

## 3. Deubiquitinating Enzymes Involved in Autophagy

Protein posttranslational modifications are crucial in the dynamic regulation of the autophagy process. Modifications of core components of the autophagic machinery are notably essential for the induction of autophagy. Autophagy proteins that are ubiquitinated constitute substrates for DUBs, therefore regulating their function and/or stability [60].

### 3.1. Regulation of Early Steps of Autophagy

#### 3.1.1. Regulation of the mTOR Complex 1 by OTUB1

**Figure 3:**
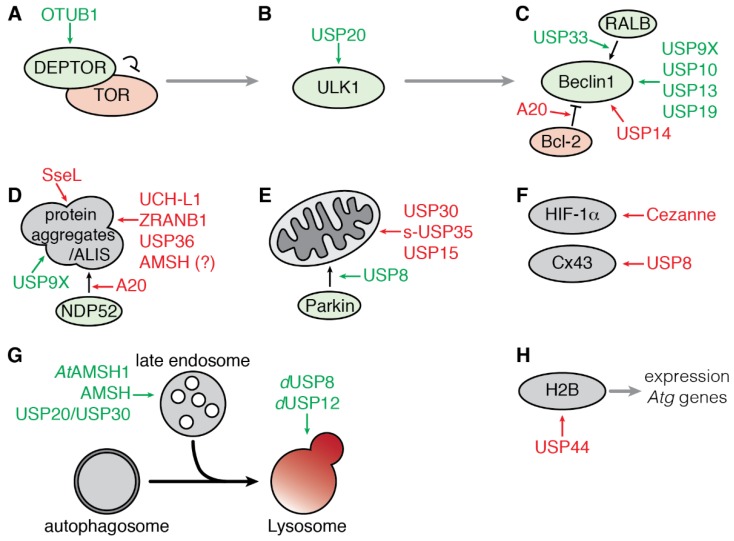
Functional roles of eukaryotic deubiquitinating enzymes in autophagy. (**A**) DEPTOR interacts with mTOR and inhibits its kinase activity. Under nutrient-rich conditions, DEPTOR is degraded continuously, and mTOR is active, promoting cell growth. However, under nutrient shortage, DEPTOR is stabilized by deubiquitination by OTUB1, interacts with mTOR and inhibits its kinase activity, allowing for the induction of autophagy. (**B**) The deubiquitination of ULK1 by USP20 allows for its stabilization and promotion of autophagy induction. (**C**) Beclin1 is an essential modulator of the PI3K-Ш complex. Beclin1 ubiquitination is crucial to its function. Deubiquitination by A20 inhibits Beclin1 activity by promoting its interaction with the inhibitor Bcl-2. USP14 negatively regulates Beclin1 activity. Other DUBs positively regulate autophagy by stabilizing Beclin1 (USP9X, USP10, USP13, USP19). The deubiquitination of RALB by USP33 benefits their interaction and the induction of autophagy. Ubiquitination also mediates the degradation of selected cargoes by autophagy. (**D**) The deubiquitination of α-synuclein aggregates by USP9X favor their degradation by autophagy, while UCH-L1 contribute to the accumulation of α-synuclein by downregulating its lysosomal degradation. The deubiquitination of various protein aggregates by ZRANB1 or USP36 impairs their degradation by autophagy. Invasion by intra-cellular bacteria tends to induce the accumulation of aggregate-like structures (ALIS) which are ubiquitinated and contribute to the induction of bacterial clearance by xenophagy; the DUB-like enzyme Ssel secreted by *Salmonella* deubiquitinates ALIS, resulting in a downregulation of autophagy and better survival of the bacteria. (**E**) Mitophagy selectively degrades mitochondria. USP8 promotes Parkin-mediated mitophagy by deubiquitinating Parkin and promoting its recruitment to the mitochondria. USP30, s-USP35, and USP15 counteract Parkin by deubiquitinating Parkin’s substrates, thus impairing mitophagy. (**F**) Specific ubiquitinated substrates can also be targeted for autophagy. Deubiquitination of HIF-1α and Connexin-43 (Cx43) by Cezanne and USP8, respectively, prevents their lysosomal degradation. (**G**) Autophagosomes can fuse with the endosomes. This fusion requires the ESCRT machinery as well as AMSH (AtAMSH1 in *A. thaliana*). USP20 and USP30 to a lesser extent favor the direction of the β2-adrenergic receptors to the autophagosome for degradation. In *Drosophila*, dUSP8 and dUSP12 positively regulate autophagy by contributing to the biogenesis of the lysosome. (**H**) Deubiquitination of H2B by USP44 negatively regulates the transcriptional activation of autophagy. Legend: Colored arrows show the action of DUBs that support (green) or inhibit (red) autophagy. Pink proteins are molecular inhibitors of autophagy; green proteins are inducers of autophagy.

The mechanistic target of Rapamycin complex 1 (mTORC1) plays a central role in the integration of various environmental signals to regulate cell metabolism, growth, proliferation, and survival. In nutrient-rich conditions, mTORC1 is active and promotes cell growth while down-regulating autophagy. Conversely, down-regulation of mTORC1 activity during nutrient deprivation activates autophagy. The protease OTUB1 was recently reported to inhibit mTORC1 activity by deubiquitinating and stabilizing the inhibitor DEPTOR in response to amino acid deprivation [61] (Figure 3A). DEPTOR stabilization by OTUB1 depends on its catalytic activity as the catalytically inactive mutant OTUB1-C91A fails to both remove ubiquitin moieties from DEPTOR and protect it from proteasomal degradation. Consistent with these observations, OTUB1 overexpression induces autophagy while its knockdown represses autophagy induction [61].

#### 3.1.2. Regulation of ULK1 by USP20

The serine/threonine protein kinase ULK1 (Unc51-like kinase 1) is a critical inducer of autophagy (see above and Figure 1). Dynamic phosphorylation and polyubiquitination regulate ULK1 activity. Notably, the ubiquitination of ULK1 with K63-linked ubiquitin chains contributes to its stabilization and activity [38]. In contrast, the linkage of K48-linked ubiquitin chains leads to proteasomal degradation of ULK1, resulting in a blockade of starvation-induced autophagy [62,63]. A loss-of-function screen of DUBs in HeLa cells identified USP20 as the first DUB to be involved in regulating ULK1 ubiquitination and stability. USP20 interacts with and deubiquitinates ULK1, thus protecting it from degradation. The maintenance of basal levels of ULK1 by USP20 contributes to rapid induction of autophagy under stress condition. Indeed, silencing of USP20 encoding gene inhibits autophagosomes and autolysosomes formation in response to starvation (Figure 3B). However, the interaction between ULK1 and USP20 is weakened upon prolonged induction of autophagy (4 to 8 h), leading to a reduction of the level of ULK1 protein while its ubiquitinated form accumulates [64]. The molecular mechanisms regulating the dissociation of USP20 and ULK1 are not known yet but could depend on posttranslational or allosteric modifications on USP20 as its stability is not affected by starvation; alternatively, the E3 ubiquitin ligase NEDD4L, may compete with USP20 to interact with ULK1 and promotes its proteasomal degradation [63].

#### 3.1.3. Regulation of the Beclin1 Complex

Beclin1 is a multivalent adaptor protein and forms, with VPS34 and VPS15, the core components of the PI3K-Ш signaling complex, which is essential for the maturation of the autophagosome (see above and Figure 1). Beclin1 also interacts transiently with accessory factors, such as ATG14L, AMBRA1, UVRAG or Bcl-2 [65]. Moreover, Beclin1 versatile ubiquitination is tightly linked to its function and activation [37]. Different types of ubiquitin chains, including K63- and K48-linked chains, were found on Beclin1, and several DUBs control Beclin1 ubiquitination status and activity (Figure 3C).

USP14 is a ubiquitin-specific protease tightly associated with the proteasome which has been shown to cleave K48-linked ubiquitin chains [66,67]; however, other studies have shown that USP14 is also able to cleave K63-linked ubiquitin chains [68,69]. Knockdown of USP14 or its inhibition with the inhibitor IU1 (see below Section 4.1) induces the activation of autophagy, indicating that USP14 is a negative regulator of autophagy in H4 (neuroglioma) cells. Because silencing the USP14 encoding gene does not affect the stability of Beclin1 or other components of the complex, this suggests that its ability to regulate autophagy is independent of its proteasomal function in H4 (neuroglioma) cells. According to this observation, it was shown that USP14 suppresses the activity of Beclin1 complex and induction of autophagy by interacting with and controlling K63- rather than K48-linked ubiquitin chains of Beclin1 [70].

Autophagy is highly interconnected with immune processes and can be triggered by activated TLR4 (Toll-like receptor 4). Activation of TLR4 by microbial components contributes to the recruitment of adaptor proteins and enzymes required for the signal transduction and induction of the immune response by NF-κB (nuclear factor kappa B) transcription factors, such as the E3 ubiquitin ligase and scaffold protein TRAF6 (TNFR-associated factor 6). TRAF6 is then responsible for the induction of autophagy following the activation of the TLR4 in macrophages through the ubiquitination of Beclin1 with K63-ubiquitin chains in murine macrophages RAW 264.7 [71]. Indeed, ubiquitination of the lysine residue 117 within the BH3 domain of Beclin1 prevents its interaction with the inhibitor Bcl-2 [71,72]. In addition, Min and colleagues showed that TRAF6 and USP14 compete for the interaction with Beclin1 in HEK293T (human embryonic kidney 293T) cells and that USP14 negatively regulates autophagy in THP-1 monocyte cells [73]. TRAF6 and Beclin1 interact through their coiled-coil domains in the absence of USP14, whereas the interaction is gradually attenuated when the cells are co-transfected with increasing quantity of USP14. However, the study does not provide any evidence for the catalytic role of USP14 and instead suggests that the interaction of Beclin1 with USP14 inhibit TRAF6-mediated ubiquitination of Beclin1 [73]. Additionally, the DUB A20—a downstream target of NF-κB—is responsible for the catalytic removal of K63-linked ubiquitin chains on the lysine 117 of Beclin1 to limit the induction of autophagy in the murine macrophage cell line RAW 264.7 [71].

Interestingly, Beclin1 and the Bcl-2 family member, MCL-1, compete for their interaction with USP9X, which contributes to their stabilization by protecting them from proteasomal degradation in HEK293T cells [74]. In the same study, it was observed that MCL-1 levels were increased in malignant tissues from melanoma patients while Beclin1 was destabilized, suggesting that USP9X promotes tumor progression. However, USP9X function in tumorigenesis appears to be more complex and context-dependent as independent studies have shown that USP9X can be either a tumor promoter or suppressor depending on the origin of the cells [75,76]. The addition of K11-linked ubiquitin chains to Beclin1 by the E3 ligase NEDD4 triggers its degradation by the proteasome [77]. The enzymes USP13 and USP19 are known to process K11-linked ubiquitin chains resulting in the stabilization of Beclin1 in HEK293T cells (for both USP13 and USP19 roles), as well as in MEF, HeLa and Bcap-37 cells (role of USP13) [78,79]. Although several lysine residues in Beclin1 may be ubiquitinated with K11-linked chains, USP19 seems to mainly target ubiquitin moieties bound to the lysine 437 of Beclin1 in HEK293T cells, suggesting that its function may not be redundant, but instead complementary to USP13 [78].

Finally, the DUB USP33 was found to be a regulator of early steps of starvation-induced autophagy activation by promoting the interaction between Beclin1 and the RAS-like GTPase RALB. Indeed, the assembly of the complex RALB-EXO84-Beclin1 is made possible by the deubiquitination of RALB at its lysine residue 47 in HEK293T and HeLa cells [80]. The identification of USP33 as indirectly regulating Beclin1 activity through the deubiquitination of one of its partners reinforces the hypothesis that the modification and activation of autophagy machinery components are context-specific.

### 3.2. Regulation of Selective Autophagy

#### 3.2.1. Aggrephagy

The formation of protein aggregates is a continuous process in the cell, and their degradation by autophagy, referred to as aggrephagy, is one of the first types of selective autophagy that has been described. Aggrephagy involves the autophagy receptors p62/SQSTM1 and NBR1 which are recruited to ubiquitinated protein aggregates [81,82,83,84].

The accumulation of protein aggregates that fail to be degraded in neuronal or glial cells is a hallmark of various neurodegenerative diseases. Aggregation of α-synuclein is characteristic of the pathogenesis of Parkinson’s disease in which mono- and poly-ubiquitinated α-synuclein are major constituents of the Lewy bodies [85,86]. Mono-ubiquitination of α-synuclein seems to be required for its targeting of proteasomal degradation and thus negatively regulates its autophagic degradation. USP9X interacts with α-synuclein in vitro and in vivo and contributes to the removal of mono-ubiquitin moieties and favors its targeting for degradation by autophagy rather than the proteasome [87]. Conversely, UCH-L1—a DUB associated with Parkinson’s disease and whose gene is frequently mutated in familial forms of the pathology [88]—promotes the accumulation of α-synuclein aggregates in oligodendrocytes [89,90]. Another independent study also reported that membrane-associated UCH-L1 contributes to α-synuclein neurotoxicity, possibly by negatively regulating its lysosomal degradation [91] (Figure 3D). Finally, a recent study demonstrates the prevalence of K63-linked ubiquitin chain conjugates in Lewy bodies, suggesting that their elimination can be primarily ensured by the lysosomal route rather than the 26S proteasome. In addition, this study identifies USP8 as one of the best markers of Lewy bodies in human pigmented neurons in sporadic cases of Parkinson’s disease and demonstrates the ability of USP8 to hydrolyze K63-linked ubiquitin chains from α-synuclein in vitro [92]. Moreover, *Usp8* gene extinction significantly reduces the total level of α-synuclein both in a *Drosophila* model of Parkinson’s disease and in human embryonic kidney (HEK293T) cultured cells [92]. Thus, the presence of USP8 on endosomal membranes (see below) and in Lewy bodies, as well as its ability to deubiquitinate α-synuclein, makes it a preferred therapeutic target according to the assumption that inhibition of USP8 could directly promote the elimination of amyloid fibers by the endocytic and lysosomal pathways.

It was recently shown that the selective autophagy receptor p62 binds to protein aggregates modified not only with K63-linked ubiquitin chains but also with K33-linked ubiquitin chains [93,94]. ZRANB1/TRABID is a K29- and K33-specific DUB [95]. Knockdown of *ZRANB1* enhances the recruitment of p62 to K33-associated protein aggregates, suggesting that ZRANB1 is a negative regulator of aggrephagy; yet, the physiological function of K33-linked ubiquitin chains and the role of ZRANB1 are not entirely understood [94].

In *Drosophila*, the p62 homolog Ref(2)P is also implicated in the clearance of ubiquitinated protein aggregates [96,97]. However, little is known about the regulation of autophagy-associated ubiquitination processes in flies. The only DUB known to regulate ubiquitin-dependent autophagy negatively is dUSP36 [98]. This protein negatively regulates the formation of ubiquitinated nuclear aggregates, while promoting cell growth. Indeed, deletion of the dUSP36 encoding gene results in the robust accumulation of ubiquitinated protein aggregates, which include the histone protein H2B, and in the activation of autophagy, independently of the TOR pathway [98] (Figure 3D).

Protein aggregates clearance requires the action of selective receptors to be adequately targeted for autophagic degradation [3]. NDP52 is an autophagy receptor mostly described for its role in addressing ubiquitin-decorated bacteria for degradation by autophagy [99,100]. However, a new role for NDP52 in the formation of TRAF6 aggregates was unveiled [101]. Indeed, NDP52 mediates the aggregation and selective autophagic degradation of the TLR adaptor molecule TRIF and the signaling molecule TRAF6 in response to TLR4 stimulation. Ubiquitination of NDP52, mediated by TRAF6, is necessary for its activity and is counteracted by A20 [101] (Figure 3D).

With the variety of proteins prone to aggregations, it is not surprising that different DUBs are involved in the regulation of the formation and degradation of protein aggregates. Protein aggregation is a hallmark of various pathologies, notably neurodegeneration and infection, and a better understanding of the regulatory mechanisms associated with each pathology will greatly benefit the development of appropriate and targeted treatments.

#### 3.2.2. Mitophagy

Mitophagy refers to the clearance of exhausted mitochondria by autophagy. The mitochondrial kinase Pink1 and the E3-ubiquitin ligase Parkin play a central role in the mitochondrial quality control. Upon mitochondria damage and loss of membrane potential, Parkin is translocated to the outer mitochondrial membrane (OMM) and activated by stabilized Pink1 [102]. Active Parkin ubiquitinates a myriad of substrates on the OMM that can be recognized by ubiquitin-binding selective autophagy receptors [103,104]. So far, three DUBs—USP30, s-USP35, and USP15—have been reported to counteract Parkin activity following acute mitochondrial depolarization, thus acting as a negative regulator of mitophagy. USP8 is the only DUB identified so far as a positive regulator of mitophagy (Figure 3E).

USP30 is a mitochondrial enzyme, tethered to the outer membrane of the mitochondria with its catalytic domain facing the cytoplasm [105]. Several independent studies point out that USP30 is one of the major DUB regulating mitophagy. Overexpression of USP30 reverses the ubiquitination of Parkin substrates, such as TOM20, and impairs mitophagy [106,107,108,109]. USP30 function in autophagy is dependent on its catalytic activity as a catalytically inactive mutant USP30-C77A is ineffective at inhibiting mitophagy [106]. The depletion of USP30 was shown to enhance the degradation of mitochondria in neuronal and HeLa cell cultures [106,109]. USP30 knockdown also increases the ubiquitination level on multiple Parkin substrates, thus confirming that USP30 antagonizes Parkin function. USP30 proteolytic activity is more efficient on K6-linked ubiquitin chains, even though it can also process K11, K48 and K63 chains [108,110]. The ubiquitin chains targeted by USP30 are similar to the ones Parkin adds to its substrates, suggesting that these two enzymes act as antagonists on shared substrates. The majority of the work carried out on the regulation of mitophagy have relied on cells overexpressing Parkin along with the use of mitochondrial-depolarizing agents. Such experiments simulate an extreme scenario of mitochondrial stress and interpretations may not be relevant to basal conditions of mitochondrial clearance [111]. However, recently published work by Marcassa and colleagues describes the investigation into the role of USP30 in more physiological conditions [112]. The authors propose a new model in which USP30 acts upstream Pink1 as the depletion of Pink1 in cells lacking USP30 abrogated the increased mitophagy induced by *USP30* knockdown in U2OS cells [112]. In the same study, USP30 is revealed to regulate the degradation of peroxisome by autophagy (pexophagy) in a similar way to its role in mitophagy. Like mitochondria, peroxisomes are the main source of reactive oxygen species (ROS) that can be damaging to the cells if produced in high quantities [113]. Moreover, contact sites between mitochondria and peroxisomes exist, and mitochondria were shown to play a role in the generation of peroxisomes [114]; thus reinforcing the hypothesis of an intrinsic relationship between both organelles. The depletion of USP30 increases both pexophagy and mitophagy. However, the localization of USP30 on mitochondria and peroxisomes relies on distinct sequences, suggesting that the role of USP30 in pexophagy is independent to its mitochondrial function [112]. It is possible that USP30 acts at different levels during the mitophagy or pexophagy processes depending on the conditions. In basal condition, USP30 could serve as a safety check-point to avoid mitophagy or pexophagy to be triggered inappropriately.

Besides, other studies identified additional DUBs which may also contribute to the regulation of mitophagy. USP35 short form (s-USP35) is another DUB that is localized at the mitochondria [109]. In a similar manner to USP30, overexpression of s-USP35 impairs mitophagy while s-USP35 knockdown enhances mitochondrial degradation [109].

USP15 is the third DUB identified to antagonize Parkin-mediated mitophagy. Overexpression of USP15 inhibits mitophagy dependently of its catalytic activity, while depletion of USP15 enhances mitophagy. Unlike USP30 and s-USP35, USP15 is only rarely localized at the mitochondria [115].

Only one DUB, USP8, may act as a positive regulator of mitophagy through Parkin regulation. *Usp8* knockdown impairs Parkin-mediated mitophagy by preventing Parkin recruitment of depolarized mitochondria. In this process, USP8 selectively removes K6-linked ubiquitin chains on Parkin and counteracts Parkin auto-ubiquitination and auto-catalytic activation [116].

Whether and how these DUBs act in concert or within different organs or situations remains to be determined to fully understand their specific requirements in physiological or stressed conditions.

#### 3.2.3. Targeted Degradation of Cargoes

Proteins can be degraded by autophagy independently of their aggregation in a way that can be either dependent or independent of their ubiquitination state. To date, only a few substrates, known to be directly targeted for degradation by autophagy in response to ubiquitination, have been shown to be regulated by a specific DUB (Figure 3F).

The Hypoxia-Inducible Factor 1, α subunit (HIF-1α) is a transcription factor essential for cells to adapt rapidly to low oxygen levels (hypoxia). When oxygen is available, HIF-1α is polyubiquitinated and rapidly degraded either by the proteasome or directly by the lysosome through chaperone-mediated autophagy [117,118]. The DUB Cezanne/OTUD7B is itself induced by oxygen deprivation in cultured endothelial cells. Moreover, loss of Cezanne reduces the amount of HIF-1α protein while Cezanne overexpression stabilizes HIF-1α and protects it from autophagic degradation in a catalytic-dependent manner by specifically processing K11-linked ubiquitin chains [119]. Mutation of the CMA-targeting motif (KFERQ motif) of HIF-1α makes it insensitive to *Cezanne* knockdown, thus suggesting that Cezanne specifically regulates the degradation of HIF-1α mediated by CMA. Cezanne is not the only DUB to regulate the ubiquitination status of HIF-1α. Indeed, USP8 is essential for the removal of ubiquitin moieties on HIF-1α in normoxia, contributing to the maintenance of a basal level of HIF-1α. In this case, however, USP8 appears to protect HIF-1α from proteasomal degradation [120]. These two studies show that different DUBs can regulate the fate of a shared substrate depending on the physiology of the cell.

Connexin-43/Cx43 is a member of the connexin family that forms the gap junction channels between adjacent cells, enabling direct intercellular exchanges between cells, which is another example of a substrate for at least two different degradative pathways. Indeed, Cx43 polyubiquitination triggers its degradation through either the proteasome or the lysosome via endocytosis and autophagy [121,122,123,124]. USP8 interacts with and deubiquitinates Cx43, removing monoubiquitin moieties as well as K63- and K48-linked ubiquitin chains. Cx43 ubiquitination and degradation by autophagy are increased in *Usp8* knockdown cells [125]. Even though USP8 regulates autophagic degradation of Cx43 in basal condition, one cannot exclude that USP8 may also affect Cx43 through the endolysosomal pathways in different conditions as USP8 is well described for its implication in the endocytosis of various plasma membrane receptors [126,127,128].

Thus, there are several cases of proteins being degraded through different processes notably as a result of the linkage of different kinds of ubiquitin moieties and undergoing tight regulation by specific DUBs.

### 3.3. Regulation of the Fusion of Endosome to Autophagosome

Autophagy and endocytosis are two conserved and interconnected degradative pathways among eukaryotes. Moreover, fusion events between autophagosomes and endocytic compartments have been observed and investigated [129,130]. Endocytosis and autophagy converge not only at the level of lysosomes but also at the level of early and late endosomes, forming another type of vesicle called amphisomes. Several DUBs, known for their role in endocytosis, also impact directly or indirectly the autophagic flux (Figure 3G).

AMSH is a metalloprotease of the JAMM type involved in the sorting of cell-surface receptors at endosomes [131,132,133,134]. AMSH localizes at the endosomes and promotes the recycling of internalized receptors [135,136]. Disruption of AMSH in mice results in the loss of neurons in the hippocampus and severe atrophy of the cerebral cortex [137]. An independent study observed that the loss of AMSH in neurons results in the accumulation of ubiquitinated protein aggregates associated with the autophagy receptor p62, indicating that the autophagy flux is impaired [138] (Figure 3D). However, at this stage of the study, it is not possible to discriminate whether the blockade of the autophagy flux results from an impairment in the endocytic process or a lack of targeting of cargoes to autophagosome, independently of endocytosis. In the plant model *Arabidopsis thaliana*, the ortholog AMSH1 interacts with the ESCRT-III protein VPS2.1 and contributes to autophagic degradation [139] (Figure 3G).

USP8 is a second DUB playing a major role in endocytosis by regulating both the ubiquitination status of cargoes and members of the ESCRT machinery regulating membrane deformation and scission events [126,127,128,140,141,142]. USP8 has been extensively studied for its role in the regulation of the trafficking and lysosomal degradation of receptors, such as EGFR, through the endocytic process [135,143]. In addition to its role in endocytosis, loss of dUSP8 in *Drosophila* blocks the progression of autophagy, resulting in the accumulation of Ref(2)P/p62 and ubiquitinated proteins [144]. As for dUSP8, dUSP12 depletion affects both the autophagic flux and endocytosis process. Indeed, silencing dUSP12 encoding gene results in the accumulation of autophagosomes in *Drosophila* [144], and USP12 negatively regulates the endocytosis and translocation of the Notch receptor to the lysosomes in both *Drosophila* and mammalian cells [145]. Although these studies cannot exclude a direct role of these DUBs in the regulation of autophagy, they support a close imbrication of endocytosis and autophagy and suggest interdependence of the two processes. This hypothesis is reinforced by the fact that disruption of the endocytic process using dominant-negative Rab or by their knockdown also results in impaired autophagy [146].

The particular case of the β2-adrenergic receptors (β2-ARs) also illustrates a reliable interconnection between the endocytic and autophagic processes and their tight regulation by ubiquitination. β2-adrenergic receptors (β2-ARs) availability on the plasma membrane is tightly regulated by balancing their internalization and recycling rates. Misregulation of β2-ARs trafficking has been associated in various pathologies, including heart failure and asthma [147]. β2-ARs take an unconventional route to the lysosomes; indeed, after their endocytic internalization, ubiquitinated β2-ARs are directed to the autophagosomes rather than the lysosomes [148]. The post-endocytic sorting of the receptor from the endosomes to the autophagosomes is modulated by the proteases USP20 and USP33 [149]. However, solely USP20 was shown to promote the deubiquitination of β2-ARs and their post-endocytic trafficking to autophagosomes. In this process, phosphorylation of USP20 at serine residue 333 is required for its activity providing an additional level of regulation [148].

Recently, the protein CHMP2A of the ESCRT-III complex was identified to be crucial for the closure of the autophagosome [150]. Therefore, endosomes-associated DUBs such as USP8 and AMSH, or other DUBs that remain to be identified, may also play a direct regulatory role in this process through the regulation of ESCRT-III components activity, as recently shown for CHMP1B during endocytosis [140].

### 3.4. Transcriptional Regulation of Autophagy by USP44

The expression of a number of genes related to autophagy is activated upon starvation in mammalian cell culture. In this process, histone protein H2B monoubiquitination (H2Bub1) is an essential modification for the regulation of gene transcription. The level of H2Bub1 is controlled by the protease USP44 which is upregulated after starvation. Knockdown of *Usp44* results in the maintenance of H2Bub1 upon starvation and abolishes the change in expression of starvation-induced autophagy-related genes. Moreover, downregulation of USP44 encoding gene blocks the induction of autophagy in mESCs (mouse embryonic stem cells) (Figure 3H). This study thus unveils a new role for DUB in the transcriptional regulation of autophagy through the modulation of H2B monoubiquitination [151].

### 3.5. Regulation of Autophagy by Bacterial and Viral DUB-Like Enzymes

Ubiquitination of microbial molecular patterns is used by eukaryotic cells to tag invasive pathogens and target them for autophagic degradation. This reaction leads to the accumulation of ubiquitinated protein aggregate known as ubiquitinated aggresome-like induced structures (ALIS). Such aggregates contribute to the upregulation of autophagy and the removal of intracellular pathogens. In response to this host defense mechanism, intracellular pathogens, such as bacteria or viruses, have developed strategies to hijack the host ubiquitin pathway by expressing DUB-like enzymes able to counteract ubiquitination and permit them to escape their elimination by autophagy.

For instance, the intracellular pathogenic bacteria *Salmonella enterica* serovar *Typhimurium* (*S. Typhimurium*) counteracts the ALIS-induced autophagy by translocating a DUB-like enzyme, SseL (*Salmonella*-secreted factor L), into the cytosol. Lysates from mouse macrophages infected with *∆sseL* mutant bacteria are enriched in ubiquitinated proteins, and immunofluorescence experiments revealed that these bacteria are more prone to ubiquitination and recognition by autophagy markers such as LC3 or p62 [152,153]. Secreted SseL deubiquitinates ALIS and the *Salmonella*-containing vacuoles, reducing the induction of autophagy, further promoting the survival and replication of *S. Typhimurium* [152] (Figure 3D).

Like *Salmonella*, *Legionella* is an intracellular bacterium that can establish niches in cytoplasmic vacuoles which allows for the survival and replication of the bacteria. The *Legionella pneumophila* effector protein RavZ is a secreted cysteine protease that interferes with the autophagy machinery by irreversibly deconjugating LC3 from the autophagosome membrane [154]. LC3 is an autophagy-related ubiquitin-like protein anchored to the autophagosome membrane. The process leading to LC3 lipidation and association to the membrane is similar to the ubiquitination process and requires a ubiquitin-like conjugation system. RavZ deconjugates LC3 from the autophagosome membrane by hydrolyzing the amide bond between the C-terminal glycine residue and an adjacent aromatic residue; the lack of terminal glycine residue prevents the conjugation of LC3 to the membrane [154,155]. RavZ can also process conventional ubiquitin chains and prevent the targeting of intracellular bacteria for autophagic degradation [156]. Indeed, using a co-infection system with *Salmonella* and *Legionella*, Kubori and colleagues showed that *Legionella* RavZ protease prevents the recruitment of the autophagy receptors p62 and NDP52 to the *Salmonella*-containing vacuoles. The lack of autophagy receptors at the SCVs is due to the removal of ubiquitin moieties from the SCVs by RavZ [156]. It was recently shown that RavZ specificity toward LC3 anchored to the autophagosomal membrane depends on its interaction with PI3P [157]; this observation suggests that RavZ activity as DUB on ubiquitin coats depends on the lipid structure of the nearby vacuole.

Autophagy also targets viruses; yet, many viruses exploit autophagy for their replication [158]. Coronaviruses induce the formation of double-membrane vesicles that allow for their replication and are often decorated with LC3 and cell infection with coronaviruses is often accompanied with induction of autophagy [159]. The non-structural protein PLP2-TM, which is a transmembrane papain-like protease with deubiquitinating activity [160], is sufficient for the accumulation of autophagosomes in different cell lines. However, its role in the regulation of autophagy is independent of its protease activity [161]. PLP2-TM interacts with LC3 and promotes the accumulation of autophagosomes by blocking their fusion with the lysosomes. In their study, Chen and colleagues suggest that PLP2-TM blocks autophagosome-lysosome fusion through its interaction with Beclin1, a prime target for viruses that manipulate the autophagy pathway [162]. PLP2-TM also promotes the interaction of STING (stimulator of interferon genes) with Beclin1, possibly to impede the activation of downstream antiviral responses, accentuated by the deubiquitination of components of the signaling cascade such as RIG-1 or TRAF3 [161,163].

These studies provide fascinating examples of possible coevolution and adaptation of the pathogens with their host, where pathogens managed to bypass the host’s defense to their own benefit.

## 4. Targeting Deubiquitinating Enzymes Acting in Autophagy for Therapeutic Purpose

Ubiquitination regulates major cellular functions by controlling protein stability and activity, and defects in this process contribute to the development of many diseases. In some cases, ubiquitin-dependent autophagic processes constitute entry points to design new treatments. Depending on the context, however, autophagy can either be beneficial and contribute to survival and recovery or have adverse effects. As such, there is a need for in-depth understanding of autophagy function and regulation in pathological or physiological situations to define in which situation the inhibition of a particular DUB will be beneficial or detrimental. Interestingly, the design of chemical tools is also a powerful strategy to probe the effect of DUBs inhibition to help both the understanding of their role in the regulation of autophagy and the design of future treatments to modulate autophagy in the corresponding pathologies.

### 4.1. The Challenge of Developing Drugs Targeting DUBs

Efficiency and usability of an inhibitor depends on its specificity. As such, the discovery of DUB-focused drugs has been challenging [20]. Indeed, although DUBs have a catalytic pocket that is suitable for drug development, their sequence and structure are very similar. Moreover, DUBs are flexible enzymes, and the regulation of their activity can involve allosteric effects as described for several DUBs that alternate between active and inactive conformations [164,165,166,167,168,169]. For instance, the free catalytic domain of USP7 undergoes significant structural modifications when it is complexed to Ubal (ubiquitin aldehyde, an irreversible DUB inhibitor) [164,170]. Recent publications of the dynamic interaction of USP7 with specific small-molecule inhibitors demonstrated that the binding of the molecules into the active site of USP7 modifies the catalytic residue C223. This modification of the active site of USP7 results in its inability to change conformation and perform the cleavage of ubiquitin chains. These studies show that the development of specific inhibitors binding to the active site of DUBs is a realistic approach, opening new avenues in the field [171,172]. In addition to intrinsic modulation of their activity and substrate specificity, some DUBs require cofactors. For instance, the full activation of USP19 requires its interaction with Hsp90, which promotes the binding of ubiquitin to the catalytic domain of USP19 [173]. Another example is the proteasome-associated enzyme USP14, whose activity is strongly enhanced when in association with the proteasome [66].

Despite the complexity of the regulation of DUBs activity, much effort has been placed in the identification and development of small-molecule regulating DUBs catalytic activity. Some of the most successful small-molecules affecting autophagy the process, through the inhibition of the activity of autophagy-associated DUBs, are shortly introduced hereafter.

### 4.2. Characterised Inhibitors of DUBs Acting in Autophagy

Screens for inhibitors of DUBs sought to identify new small-molecules with potential in two main therapeutic fields, for cancer treatment and neurodegeneration (reviewed in [20]). In both fields, some of the inhibitors’ targets play a role in the regulation of autophagy that possibly contributes to pathogenesis.

USP14 is an enzyme associated with the proteasome, which plays an essential role in the regulation of protein turnover. The role of USP14 is particularly important in neurons to maintain synaptic functions and constitutes an appealing target for drug development in order to modulate the activity of the proteasome [174]. A screen of 63,052 compounds using proteasome reconstituted with USP14, led to the identification of the first inhibitor of USP14. The small-molecule IU1 inhibits specifically USP14 with an IC_50_ of 4–5 µM [66]. The inhibitor IU1 blocks the activity of USP14 only in the presence of the proteasome, suggesting that it binds only to the activated enzyme. Moreover, the compound IU1 abrogates the catalytic activity of USP14 without affecting its noncatalytic regulatory function [66]. However, with the growing number of USP14 substrates identified, there was a need for the development of IU1 analogs with improved selectivity over the USP14-substrate complexes. A curated screen of 87 variants of IU1 led to the identification of IU1-47 as a new potent inhibitor of USP14. IU1-47 treatment of murine primary neuron cultures and in neurons derived from human-induced pluripotent stem cells (iPSC) accelerates the degradation of the microtubule-associated protein Tau, which is implicated in many neurodegenerative diseases. Besides, the inhibition of USP14 by IU1-47 induced an increase of the autophagy flux, consistent with the increased degradation rate of Tau [175].

As mentioned above, UCH-L1 is a negative regulator of autophagy widely studied for its implication in Parkinson’s disease and its contribution to the aggregation of α-synuclein as a result of autophagy blockade [86,89]. UCH-L1 is also expressed in various primary lung tumors while not detectable in normal, healthy lung tissue, suggesting a possible contribution to cancer [176]. Because of the correlation between UCH-L1 and tumor progression, as well as its implication in neurodegenerative disease, UCH-L1 is a recognized target for the development of therapeutic inhibitors. As such, the compound LDN-57444 was identified in a high throughput drug screening as a specific UCH-L1 inhibitor. Treating H1299 lung cancer cell line with this compound significantly reduces the cell proliferation rate [177]. NSC632839 is another inhibitor that affects UCH-L1 activity. However, NSC632839 activity is not specific to UCH-L1, and it is already known to inhibit USP2 and USP7 [178,179]. The amount of p62 in cells is reduced after treatment with both LDN-57444 and NCS632839, suggesting that these drugs could prevent the accumulation of protein aggregates [178]. Therefore, these inhibitors may constitute new tools to investigate further the implication of UCH-L1 in Parkinson’s disease and evaluate whether UCH-L1 inhibition favors the clearance of α-synuclein aggregates in neurons.

Inhibition of early regulators is another strategy to inhibit autophagy in some situations. The inhibitor WP1130, which targets USP9X, was reported to lead to an increase in ULK1 ubiquitination, inducing its transfer to the aggresomes and its inhibition, further resulting in the blockade of autophagy in several cultured cell lines, including the bone osteosarcoma U2OS cell line [180]. It was speculated that the inhibition of USP9X could be responsible for the accumulation and subsequent aggregation of ubiquitinated ULK1. However, silencing *Usp9X* did not result in changes in ULK1 expression level when cells were treated with WP1130. This could be because WP1130 is only partially specific and could have other targets in vivo that remain to be discovered [180,181].

In order to screen and select new small-molecules interfering with autophagy in mammalian cells, an imaging-based assay has been optimized by Liu and colleagues that makes use of cells expressing the autophagy marker GFP-LC3 to quantify the accumulation of autophagosomes [79]. Using this assay, they screened the ICCB known bioactives library, a collection of 472 compounds, and they identified the inhibitor spautin-1 (specific and potent autophagy inhibitor 1). Spautin-1 inhibits the catalytic activity of both USP10 and USP13 with an IC_50_ of ~0.6–0.7 µM. These two DUBs are involved in the regulation of Beclin1 ubiquitination in the Vps34 complex and, therefore, constitute an entry point to modulate the initiation of autophagy. Cancer cell lines treated with spautin-1 demonstrated an increased cell death rate under starvation conditions. As such, spautin-1 constitutes a potential lead for the development of autophagy inhibitors for anti-cancer therapies [79].

Because of its essential function in mitophagy, which is crucial to clear damaged mitochondria notably in neuronal cells, several small-molecule inhibitors of USP30 have been developed in the past few years. For example, based on a phenotypic screening, it was shown that the inhibition of USP30 by the compound 15-oxospiramilactone enhances the activity of USP30’s targets Mfn1 and Mfn2—two GTPases anchored at the OMM and essential for tethering adjacent mitochondria—and promotes mitochondrial fusion, thus contributing to the restoration of the mitochondria network [182]. More recently, an in vitro study identified a new small-molecule MF-094, as a potent and selective inhibitor of USP30. This compound has the opposite effect of 15-oxospiramilactone, as MF-094-mediated inhibition of USP30 accelerates mitophagy [183]. These two studies highlight the fact that the same DUBs can be involved in different processes, dependent on the signal they may receive and the interaction within different protein complexes.

There is no doubt that new inhibitors of DUBs will arise with problems related to the existence of several substrates or to poor selectivity, requiring in-depth analysis of the selected compounds in different cell types and stress situations before any preclinical assays. Interestingly, these investigations may tell a lot about how DUBs regulate autophagy and other cell processes, and may be used as molecular tools to unveil regulatory mechanisms.

## 5. Conclusions

Protein ubiquitination is an essential, reversible, posttranslational modification involved in virtually every cellular process. The past decades have seen remarkable progress in the understanding of the function of DUBs, their mechanism of action and regulation. Recently, there has been an increasing body of evidence that ubiquitination plays a crucial role in regulating autophagy, and DUBs intervene at multiple steps in autophagy. Deregulation in both autophagy and ubiquitination/deubiquitination processes have been linked to many pathologies such as neurodegenerative diseases, cancer onset and progression, and different kinds of viral or bacterial infections. Also, considerable effort was placed on the development and optimization of small-molecules acting as DUBs inhibitors. Such molecules can serve not only as leads for the development of drug-like molecules but also as tremendous useful tools to investigate the molecular mechanism of autophagy and its regulation by the ubiquitin system. By the development of molecules targeting protein-protein interaction instead of the catalytic activity, it could be possible to manipulate and orientate precisely the function of a DUB towards a given process and/or target to avoid pleiotropic effects.
